# Home-based transcranial alternating current stimulation (tACS) in Alzheimer’s disease: rationale and study design

**DOI:** 10.1186/s13195-023-01297-4

**Published:** 2023-09-15

**Authors:** Daniele Altomare, Alberto Benussi, Valentina Cantoni, Enrico Premi, Jasmine Rivolta, Chiara Cupidi, Alessandro Martorana, Emiliano Santarnecchi, Alessandro Padovani, Giacomo Koch, Barbara Borroni

**Affiliations:** 1 Department of Clinical and Experimental Sciences, Neurology Unit, University of Brescia, Brescia, Italy; 2 Department of Continuity of Care and Frailty, ASST Spedali Civili, Piazzale Spedali Civili 1, 25123 Brescia, Italy; 3 Stroke Unit, ASST Spedali Civili, Brescia, Italy; 4 Neurology Unit, Fondazione Istituto G. Giglio, Cefalù, Italy; 5 Department of Systems Medicine, Memory Clinic, University of Rome Tor Vergata, Rome, Italy; 6 Department of Radiology, Precision Neuroscience and Neuromodulation Program, Gordon Center for Medical Imaging, Massachusetts General Hospital, Harvard Medical School, Boston, USA; 7 Department of Clinical and Behavioral Neurology, Fondazione Santa Lucia IRCCS, Rome, Italy; 8 Department of Neuroscience and Rehabilitation, University of Ferrara, Ferrara, Italy

**Keywords:** Alzheimer’s disease, Electrical stimulation, tACS, Biomarkers, Cognition, Memory

## Abstract

**Background:**

Gamma (γ) brain oscillations are dysregulated in Alzheimer’s disease (AD) and can be modulated using transcranial alternating stimulation (tACS). In the present paper, we describe the rationale and design of a study assessing safety, feasibility, clinical and biological efficacy, and predictors of outcome of a home-based intervention consisting of γ-tACS over the precuneus.

**Methods:**

In a first phase, 60 AD patients will be randomized into two arms: ARM1, 8-week precuneus γ-tACS (frequency: 40 Hz, intensity: 2 mA, duration: 5 60-min sessions/week); and ARM2, 8-week sham tACS (same parameters as the real γ-tACS, with the current being discontinued 5 s after the beginning of the stimulation). In a second phase, all participants will receive 8-week γ-tACS (same parameters as the real γ-tACS in the first phase). The study outcomes will be collected at several timepoints throughout the study duration and include information on safety and feasibility, neuropsychological assessment, blood sampling, electroencephalography, transcranial magnetic stimulation neurotransmitter measures, and magnetic resonance imaging or amyloid positron emission tomography.

**Results:**

We expect that this intervention is safe and feasible and results in the improvement of cognition, entrainment of gamma oscillations, increased functional connectivity, reduction of pathological burden, and increased cholinergic transmission.

**Conclusions:**

If our expected results are achieved, home-based interventions using γ-tACS, either alone or in combination with other therapies, may become a reality for treating AD.

**Trial registration:**

PNRR-POC-2022–12376021.

## Background and rationale

Alzheimer’s disease (AD) is the first cause of dementia worldwide, and its prevalence is expected to triple by 2050 resulting in massive burden and costs for patients, families, and society [1]. Hence, interventions aimed at preventing or at least delaying the disease progression are urgently needed. A large body of research has focused on the development of disease-modifying drugs acting on AD pathophysiological mechanisms [2]. Among them, amyloid deposition is the most common target [2], consistently with the amyloid hypothesis [3, 4]. Recently, the first anti-amyloid monoclonal antibodies, aducanumab [5] and lecanemab [6], have been approved by the Food and Drug Administration (FDA) in the USA under the accelerated approval pathway, while others are currently under review. Although the clinical efficacy of anti-amyloid monoclonal antibodies is yet to be proven, it has been estimated that aducanumab would only be suitable for 1–12% of the real-word memory clinic population [7, 8]. Therefore, it is necessary to continue research into alternative therapeutic approaches targeting different mechanisms of action.

An increasing body of evidence suggests that gamma (γ) brain oscillations (typically 30–100 Hz) play a crucial, even though not fully understood, role in AD [9]. Gamma oscillations are prominent across multiple brain regions including the hippocampus [10–12], and are involved in various higher brain functions including long-term memory, working memory, attention, and sensory processing [13], and are involved in neural communication and synaptic plasticity [13]. Moreover, gamma oscillations typically emerge from the coordinated interaction of brain excitation and inhibition [14]. Indeed, gamma oscillations are generated by parvalbumin fast-spiking interneurons: GABAergic cells (with inhibitory effects) that play a major role in the hippocampus and in maintaining a fine-tuned excitation-inhibition balance in the brain [15]. Dysregulation of gamma oscillations has been observed in AD [16]. Moreover, the excitation-inhibition balance is commonly disrupted in AD, in which parvalbumin fast-spiking interneurons are particularly vulnerable and their decrease in the hippocampus might contribute to AD pathogenesis via an increase in glutamatergic excitotoxicity in the hippocampus [15]. This might contribute to the cognitive deficits and synaptic dysfunction commonly observed in this disease. Indeed, synaptic dysfunction, along with amyloid and tau deposition, is an early event in AD pathophysiology, preceding neurodegeneration [17–19]. Therefore, we hypothesize that the regulation of the gamma rhythm might improve AD symptoms and pathophysiology.

A promising approach for modulating brain oscillations is transcranial alternating current stimulation (tACS). tACS is a non-invasive brain stimulation (NIBS) technique that uses a low-intensity alternating (sinusoidal) electrical current to modulate brain activity by entraining specific cortical rhythms based on the applied stimulation frequency. The use of tACS to entrain gamma oscillations (γ-tACS) in AD patients is a promising avenue of research, as it has the potential to restore normal brain function and improve cognition through physiological mechanisms related to gamma modulation, including modulation of neuroplasticity. The precuneus might be an ideal target for γ-tACS as it is the main node of the default mode network (DMN, a network of brain regions that is active when the brain is at rest and not engaged in a specific task) [20] and is also involved in other large-scale brain networks [21], is involved in a wide range of cognitive processes including episodic memory [22], and has been shown to be particularly affected in AD [23, 24]. The DMN has been shown to be disrupted in AD [23, 25], and restoring normal activity in this network has been proposed as a potential therapeutic strategy.

A major limiting factor of most NIBS protocols is that they require multiple stimulation sessions to induce cumulative and long-lasting aftereffects, and are often implemented in a clinical setting, requiring participants to travel to a hospital or clinic. This can prevent the administration of long-duration interventions, especially for patients with neurodegenerative disorders. This issue might be overcome by implementing γ-tACS in a home setting.

This is the rationale for the *Brain Synchronization to Treat Early-stage Alzheimer’s Disease* (BrainSync-AD) study. BrainSync-AD aims to assess the safety, feasibility, clinical and biological efficacy, and predictors of outcome of a home-based intervention consisting of precuneus γ-tACS. In the following sections, we outline the study design and expected results of BrainSync-AD.

## Study design

**Fig. 1:**
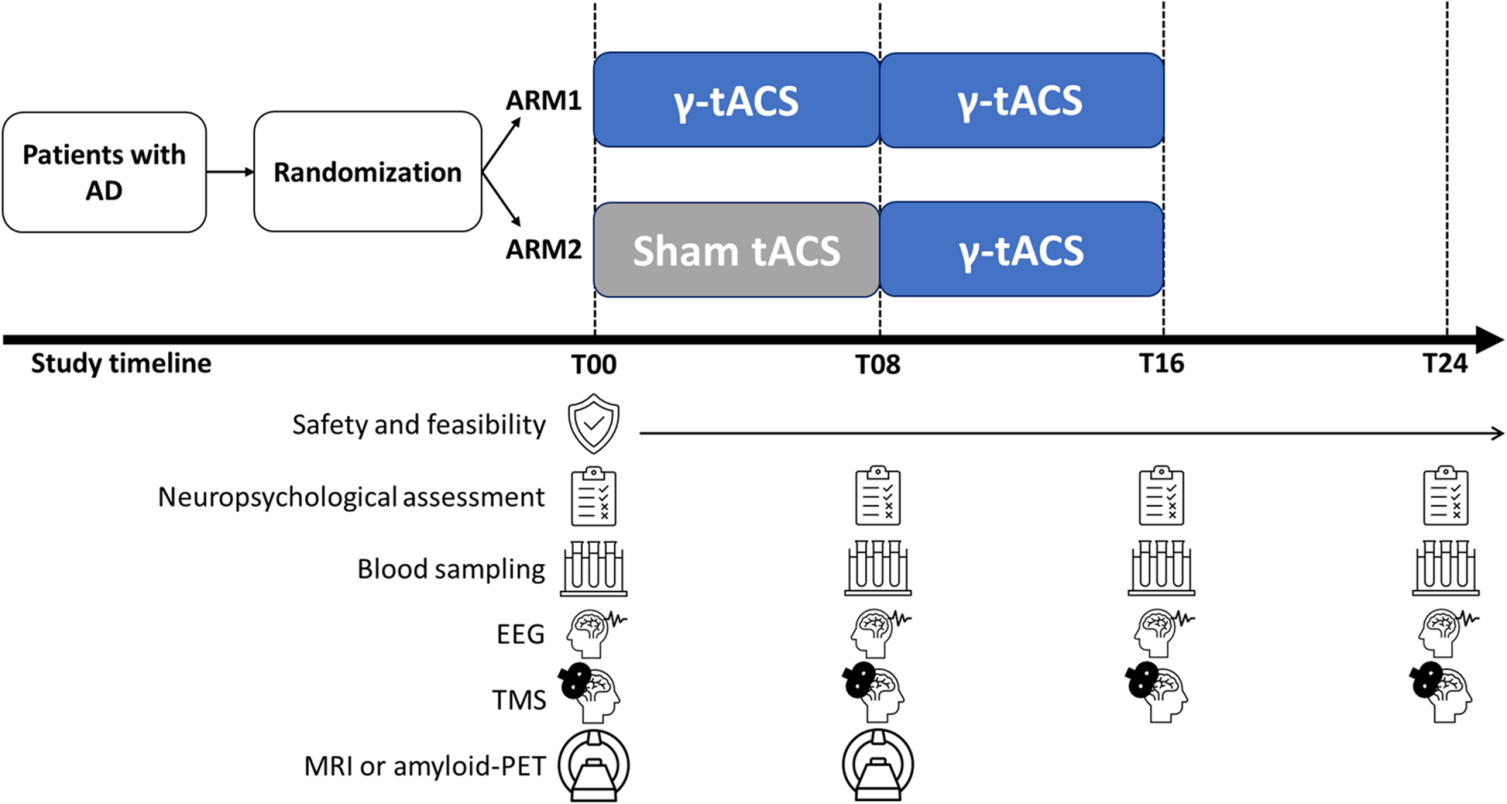
BrainSync-AD study design AD, Alzheimer’s disease; γ, gamma; tACS, transcranial alternating current stimulation; EEG, electroencephalography; TMS, transcranial magnetic stimulation; MRI, magnetic resonance imaging; PET, positron emission tomography; T00, baseline; T08, 8-week follow-up; T16, 16-week follow-up; T24, 24-week follow-up. Safety and feasibility will be constantly monitored throughout the duration of the study. Neuropsychological assessment, blood sampling, EEG, and TMS will be collected at T00, T08, T16, and T24. MRI and amyloid-PET will be collected at T00 and T08

BrainSync-AD is a multicenter study consisting of two phases (Fig. 1). In the first double-blind, randomized, placebo-controlled phase, participants will be randomized in a 1:1 ratio into two study arms: ARM1, where participants will receive 8-week real γ-tACS over the precuneus; and ARM2, where participants will receive 8-week sham tACS over the precuneus. In the second open-label phase, all participants will receive 8-week real γ-tACS over the precuneus. Each participant will receive a total of 16 weeks of intervention (8 weeks of real or sham tACS depending on the randomization, and 8 weeks of real tACS) with 5 tACS sessions per week (Monday–Friday), each session lasting 1 h. Differently from alternative study designs (e.g., a simple randomized placebo-controlled or a crossover), our study design allows all participants to benefit from the experimental intervention (unlike in a simple randomized placebo-controlled study where controls do not undergo the experimental intervention) yet providing a control condition (T00–T08 in ARM2), and to compare the efficacy of a longer (ARM1, 16 weeks) and of a shorter (ARM2, 8 weeks) experimental intervention (unlike in a crossover study where the duration of the experimental intervention is the same for all participants).

Visits will be conducted at baseline (T00) and after 8 (T08, end of the double-blind and beginning of the open-label phase), 16 (T16, end of the open-label phase), and 24 (T24, follow-up) weeks. At each visit, participants will undergo a comprehensive clinical and neuropsychological assessment, blood sampling, electroencephalogram (EEG), and transcranial magnetic stimulation (TMS) measures. At T00 and T08, participants will undergo magnetic resonance imaging (MRI) or amyloid positron emission tomography (PET) scans. The occurrence of adverse events as well as participants’ and study partners’ compliance and feedback will be monitored throughout the duration of the study. The study will be supervised by an external advisor (ES).

### Participants and setting

Participants will be 60 consecutive memory clinic patients with an etiological diagnosis of AD [26, 27] supported by evidence of AD pathology (i.e., cerebrospinal fluid Aβ_1–42_ ≤ 600 ng/L and tau ≥ 400 ng/L, or positive amyloid-PET) and Mini-Mental State Examination ≥ 19. Therefore, participants might be either in the MCI or in the dementia stage. Participants will be recruited from two centers: (i) the Neurology Unit, University of Brescia, Italy; and (ii) the Neurology Unit, Fondazione Istituto G. Giglio, Cefalù, Italy. The presence and active participation of a study partner (e.g., caregiver) is a requirement for inclusion in the study and will be carefully verified before enrollment.

Exclusion criteria include cerebrovascular disorders, hydrocephalus, intracranial masses documented by MRI, a history of traumatic brain injury, serious medical illness other than AD, a history of seizures, and the presence of electronic (e.g., pacemaker) or metal implants in the head.

### Endpoints

The primary endpoints of the study are: *i)* safety and feasibility, and *ii)* clinical efficacy of a home-based intervention consisting of γ-tACS in AD patients.

i. *Safety and feasibility*. Safety will be measured in terms of frequency and severity of adverse events, and feasibility will be evaluated based on attrition rate through remote control of the devices and supervision of the sessions. Safety and feasibility will be closely monitored throughout the study.
ii. *Clinical efficacy*. Clinical efficacy is defined as changes in clinical measures and cognition (with a focus on global cognition and individual cognitive functions, see the “Clinical and neuropsychological assessment” section for more details). Clinical Dementia Rating (CDR) sum of boxes (SB) will be the main clinical efficacy outcome. At T08, the clinical efficacy outcomes will be compared between participants who received real γ-tACS (ARM1) and those who received sham tACS (ARM2). At T16, the clinical efficacy outcomes will be compared between participants who repeated 8-week real γ-tACS interventions (ARM1) and those who received a single 8-week real γ-tACS intervention (ARM2). At T24, the long-lasting (i.e., after 8 weeks after the conclusion of the intervention) clinical effects of γ-tACS will be evaluated for both ARM1 and ARM2.

As secondary endpoints, we will assess (*iii*) biological efficacy and (*iv*) predictors of clinical and biological efficacy.

iii. *Biological efficacy*. Biological efficacy will be evaluated by changes in brain entrainment (specifically in the gamma band) as assessed by EEG (see the “EEG collection and analyses” section), changes in structural and functional brain connectivity using MRI (see the “MRI collection and analyses” section), changes in AD biomarkers measured through amyloid-PET (see the “Amyloid-PET collection and analysis” section) and blood sampling (see the “Blood-based biomarkers collection and analysis” section), and changes in cholinergic neurotransmission as measured indirectly through transcranial magnetic stimulation (TMS) (specifically through short-latency afferent inhibition (SAI), see the “TMS measures” section). At T08, the biological efficacy outcomes will be compared between participants who received real γ-tACS (ARM1) and those who received sham tACS (ARM2). At T16, the biological efficacy outcomes will be compared between participants who repeated 8-week real γ-tACS interventions (ARM1) and those who received a single 8-week real γ-tACS intervention (ARM2). At T24, the long-lasting (i.e., 8 weeks after the conclusion of the intervention) biological effects of γ-tACS will be evaluated for both ARM1 and ARM2.
iv. *Predictors of clinical and biological efficacy*. We will investigate whether demographics variables, *Apolipoprotein E* (*APOE*) genotype, the clinical and cognitive stage, and biomarkers are associated with better response to treatment. Based on previous research, we expect higher efficacy in *APOE*ε4 non-carriers [28].

### Procedures

#### tACS parameters, administration, and monitoring

##### tACS parameters

tACS will be delivered using mini-CT, a Soterix Medical device, through a pair of saline-soaked (0.9% NaCI) surface sponge electrodes placed on the scalp over the precuneus (centered over the Pz position according to the 10–20 international EEG coordinates) and over the right deltoid muscle. A custom headset, which is personalized for each participant, has been developed by the BrainSync-AD team to selectively stimulate specific brain areas. The electrodes will be secured using elastic gauzes and electroconductive gel will be applied to electrodes to reduce contact impedance (< 5 kΩ for all sessions). During real stimulation, a sinusoidal current of 2 mA will be constantly applied for 60 min. In the sham stimulation, the current will be discontinued 5 s after the beginning of the stimulation to make this condition indistinguishable from the real stimulation.

##### tACS administration

Before the beginning of the stimulation period, participants will receive a list of codes necessary to initiate the stimulation sessions. Participants will be instructed to enter these codes, one for each session, into the tACS device in order to initiate the session. Each code can only be used once. During the first sessions of stimulation, participants will receive in-person hands-on training in hospital, irrespective of randomization. During this training, an experienced member of the study team will carefully explain all the procedures (including participant’s preparation, electrodes’ placement, proper use of the custom headset, and device setup), and the study partner will be able to practice and administer the first tACS sessions under direct supervision. Once the study partner is able to complete all the procedures (as assessed through a skills test), they will be allowed to conduct the following sessions at home. We expect this in-person training to last, on average, 5 sessions. However, the training period might last longer at the participant/study partner’s request (e.g., if they are not comfortable with the procedures), or if the study team deems it appropriate. After the conclusion of the in-person training, tACS sessions will be conducted at the participant’s home with the active participation of the study partner. Participants are required to complete a minimum of 80% of the tACS sessions during the study period. Each tACS session will be considered valid if at least 95% of the session (i.e., 57 out of 60 min) is conducted without any critical events (e.g., high impedance occurring).

##### tACS monitoring

The study team will monitor the tACS sessions remotely through the verification of the codes entered by participants before the beginning of each session and through video calls. During these video calls, the study team will ensure that the electrodes are properly placed and that the device is being used correctly (e.g., turning it on and off and entering the code). The tACS device is programmed to work (i.e., deliver the stimulation) only once a day and within a specific time window (from 9am to 5pm), to ensure that the study team is always available during the stimulation sessions. The use of codes and the device settings will allow the study team to keep track of the number of tACS sessions that are administered and to assess the validity of each session. These measures help ensure the proper administration of tACS. A member of the study team will be available 24/7 via a dedicated telephone number for eventual concerns or problems (e.g., occurrence of adverse events).

#### Clinical and neuropsychological assessment

At baseline, sociodemographic and clinical information will be collected, including education level, comorbidities, and family history of neurological/neurodegenerative disorders.

A comprehensive clinical and neuropsychological battery will be administered to all participants at T00, T08, T16, and T24. This battery includes tests assessing disease severity (CDR-SB), global cognition (Alzheimer’s Disease Assessment Scale – Cognitive Subscale), associative memory (face-name association test), episodic memory (Rey Auditory Verbal Learning Test, Immediate and Delayed Recall), executive functions (verbal fluency; and Trail Making Test), independence in basic and instrumental activities of daily living (Alzheimer’s Disease Cooperative Study Activities Daily Living), behavioral disturbances (Neuropsychiatric Inventory – Questionnaire), and caregiver burden (Caregiver Burden Inventory).

#### EEG collection and analyses

An EEG recording will be conducted using a 64-channel EEG system, with a sampling rate of 1000 Hz. Both time–frequency and coherence analysis will be performed to identify the absolute and relative power of each frequency band (delta, theta, alpha, sigma, beta, and gamma) and to measure the synchronization between EEG signals, which is considered as a representation of their functional interaction. Data will be analyzed using the EEGLAB toolbox. The EEG segment will be divided into 2-s epochs. Raw data will be processed to eliminate artifacts from neurophysiological signals through visual inspection and independent component analysis. Specifically, focal and brief high power-high frequency components will be isolated and removed through an independent component analysis in order to prevent possible contamination of the gamma-band with muscle high-frequency activity. The power of the frequency bands will be quantified for each epoch through a Fast Fourier Transform, and both the mean absolute and the mean relative powers of each frequency band will be calculated. The temporal power variations at T08, T16, and T24 will be compared to T00. For network connectivity analysis, the time–frequency representation will be transformed, and the strength of network interactions will be estimated through coherence calculation. Coherence measures the synchrony between signals from different electrodes at each frequency range, and is widely used to determine if different areas of the brain are generating signals that are significantly correlated (coherent) or not (not coherent). If the signals measured by two electrodes are identical, the coherence value will be 1, and it will approach 0 as the signals become dissimilar. With this method, we will evaluate if the effect of γ stimulation is restricted to the area of stimulation or if it restores network connectivity between areas that are affected in AD. Additional coupling measures will be used to complement the coherence analysis.

#### MRI collection and analyses

A subgroup of participants (*n* = 40) enrolled in Brescia will undergo MRI scans at T00 and T08. The MRI scans will be conducted following a standard acquisition protocol [29] using a 3T scanner (Siemens Skyra) and will include T1-weighted magnetization-prepared rapid gradient echo (MPRAGE) images, diffusion-weighted images, T2-weighted echo planar imaging sequences sensitized to blood oxygenation level-dependent contrast for resting-state functional MRI (rs-fMRI), and arterial spin labeling (ASL).

Briefly, the structural analysis of gray matter will be conducted using MPRAGE images with volume and thickness calculated using Statistical Parametric Mapping (SPM12, v.7219) [30] and CAT12 (v.1742) [31]. Diffusion-weighted images will be preprocessed using the Data Processing & Analysis for Brain Imaging (DPABI) [32] toolbox for diffusion tensor imaging analysis of white matter structural data. Several approaches will be applied on rs-fMRI data (for example, using SPM12, DPABI, and GIFT toolbox [33]) to examine local connectivity [34], large-scale networks [35], and dynamic functional brain connectivity [36, 37] perturbation primarily related to the DMN. Moreover, the graph connectivity approach (e.g., BRAPH 2.0 toolbox [38]) will be interesting to confirm local (i.e., DMN-related) as well as at-distance perturbation of functional connectivity due to γ-tACS. In addition, single- and multi-label ASL will be performed to assess changes in brain perfusion using a perfusion-weighted FQ2T sequence. Quantitative cerebral blood flow (CBF) (mL/100g/min) will be estimated and measured as a perfusion index using FSL BASIL toolset [39], with the analysis focused on DMN regions to quantify the difference in absolute CBF before and after treatment.

#### Amyloid-PET collection and analysis

A subgroup of participants (*n* = 20) enrolled in Cefalù will undergo amyloid-PET scans at T00 and T08. The scans will be acquired using 18F-florbetaben following a standard acquisition protocol [40] using a GE Discovery STE 8 Slice PET/CT scanner. Patients will receive an intravenous injection of 296 MBq ± 10% of 18F-florbetaben. The dose will be administered as a single bolus injection followed by 20 cc of saline flush. Patients will rest in a waiting room after the 18F-florbetaben injection. Image acquisition will start approximately 90 min after injection, with a scan duration of 15 min. Once ready for the study, the patient will be positioned in the scanner. A CT scan (helical, kV = 120 kV, mA = 20, reconstructed slice thickness = 2.5 mm) will be acquired to be used for attenuation correction of PET data. 3-Dimensional (3D) PET acquisition (list mode) will start 90 min after 18F-florbetaben injection. Image reconstruction will be performed by using a 3D-OSEM algorithm with the following parameters: image matrix = 128, field of view = 250 mm, subsets = 24, iterations = 3, post filter = 3 mm full width half maximum, attenuation correction = CT-based. Amyloid-PET scans will be visually assessed by a trained local nuclear medicine physician using an FDA/EMA-approved reading method. A composite cortical standardized uptake value ratio (SUVr) will be computed. The change in regional SUVr over time will be assessed using a simple subtraction (SUVr follow-up − SUVr baseline) and as a percent change ([SUVr follow-up − SUVr baseline]/SUVr baseline] × 100).

#### Blood-based biomarkers collection and analysis

Plasma will be collected through venipuncture, processed, and stored at − 80°C according to standardized procedures, in each patient and at each time-point. Blood biomarkers of amyloidosis (i.e., Aβ_42_ and Aβ_42_/Aβ_40_), tauopathy (i.e., p-tau), neurodegeneration (i.e., neurofilament light), astrogliosis (i.e., glial fibrillary acidic protein), and synaptic plasticity (i.e., neurogranin) will be evaluated using commercial assays (Simoa Technology, Quanterix), following the manufacturer’s instructions.

#### TMS measures

A TMS figure-of-eight coil (each loop diameter 70 mm) connected to a monophasic Magstim Bistim [2] system (Magstim Company, Oxford, UK) will be employed, as previously reported [41]. The resting motor threshold will be determined on the left motor cortex as the minimum intensity of the stimulator required to elicit motor evoked potentials with an amplitude of 50 μV in 50% of 10 consecutive trails, recorded from the right first dorsal interosseous muscle during full muscle relaxation.

SAI, an indirect measure of cholinergic neurotransmission, will be studied using a paired-pulse model, employing a conditioning-test design. The test stimulus will be adjusted to evoke motor evoked potentials of approximately 1 mv amplitude in the relaxed right first dorsal interosseous muscle. SAI will be evaluated employing a conditioning stimulus of single electrical pulses (200 μs) delivered at the right median nerve at the wrist, using a bipolar electrode with the cathode positioned proximally, at an intensity sufficient to evoke a visible twitch of the thenar muscles [42]. Different interstimulus intervals (− 4, 0, + 4, + 8 ms) will be fixed relative to the N20 component latency of the somatosensory evoked potential of the median nerve. For each interstimulus interval, ten different paired conditioning-test stimuli and fourteen control test stimuli will be delivered to all participants in a pseudo-randomized sequence, with an inter-trial interval of 5 s (± 10%). SAI has been proposed as a diagnostic marker because of its ability to accurately detect AD and to differentiate it from FTD [41, 43–46].

### Data handling and record keeping

Data will be pseudo-anonymized, and each participant will be assigned a unique identifier. All data collected during the study will be stored on Research Electronic Data Capture (REDCap), a secure web application for building and managing online surveys and databases that comply with international standards [47], supervised by the Biostatistical and Bioinformatics Office of the University of Brescia. To ensure blindness, the information on randomization will not be stored in REDCap.

### Ethical and regulatory considerations

The study will be conducted in accordance with the International Conference on Harmonization/WHO Good Clinical Practice standards (www.ema.europa.eu/en/ich-e6-r2-good-clinical-practice). Ethical approval for the study will be obtained from both the coordinating center and local ethical review committees. Participants will provide informed consent for all aspects of the study. Agreements for transferring clinical data and biological samples will also be put in place.

## Discussion and expected results and impact

There is a growing body of evidence supporting the use of NIBS techniques for treating patients with various pathological conditions, including AD. The most commonly used NIBS techniques include repetitive TMS (rTMS) and transcranial electrical stimulation. rTMS induces a highly localized electric current that is strong enough to determine cortical excitability via membrane polarization. In contrast, electrical stimulation techniques produce a much weaker and less focalized electric field that can only modulate, but not determine, cortical excitability. Both rTMS and electrical stimulation can induce long-lasting depression or potentiation aftereffects on cortical excitability.

Recent studies on AD patients have shown that stimulation of the precuneus using rTMS has positive effects on episodic memory [48], global cognition [49], and independence in the daily living activities [49], and led to the enhancement of brain oscillations in the gamma [49] and beta [48] bands, increased neural activity in the precuneus [48], and modification of functional connections between the precuneus and the medial frontal areas [48]. These findings support the crucial role of the precuneus in AD-related memory impairment and pathophysiology.

While rTMS may have a greater clinical impact compared to electrical stimulation techniques [50], it requires expensive equipment and must be administered by dedicated expert personnel in hospital settings. This limits its widespread use for clinical populations, particularly for patients with neurodegenerative disorders who often have limited compliance. In light of this, tACS has recently drawn the attention of the scientific community thanks to its ability to entrain gamma oscillations, commonly impaired in AD, and to its low cost and potential for home-based application.

Nevertheless, the neural mechanisms behind the enhancement of gamma oscillations and its effects on cognition and other biological measures (such as AD biomarkers and neurotransmission) in AD require further investigation. Recent studies in AD have shown that γ-tACS over the precuneus can improve memory performance [28, 51] and restore cholinergic transmission [28, 51], while γ-tACS targeting temporal regions can increase blood perfusion [52] and reduce tau burden [53] in these regions.

From a biological perspective, in the present study, we expect that multi-session of γ-tACS will lead to enhanced power of fast brain oscillations (mostly gamma but possibly also beta) counterbalanced by reduced power of the slow ones (e.g., theta), not limited to the precuneus but also spreading to other structurally and functionally connected brain areas, such as those involved in the DMN or in other brain networks. The stimulation of precuneus is also expected to result in increased functional connectivity within the DMN, and possibly also in other brain networks. The perturbation of the DMN connectivity could represent an interesting surrogate marker for the effect of γ-tACS. In particular, we could be able to observe an enhancement of large-scale network connectivity of the DMN as well as a modulation of the relationship between the DMN and other large-scale networks (using dynamic functional network connectivity analysis), to further support the whole-brain effect of γ-tACS. In regard to AD biomarkers, while previous studies have observed a reduction of amyloid burden associated with gamma entrainment using sensory stimulation in mouse models of AD [54, 55], a previous pilot study found that γ-tACS applied to the bitemporal lobes reduced tau burden but not amyloid burden in patients with mild-to-moderate AD [53]. An increase in cholinergic transmission is also expected, consistent with previous findings [28, 51]. As acetylcholine is an excitatory neurotransmitter, this might contribute to restore the balance of excitation and inhibition.

Taken together, the combined biological effects of γ-tACS may lead to improved (or at least preserved) cognition. Due to the specific effect of γ-tACS on AD pathophysiology, we expect a larger effect on memory (and therefore global cognition) rather than other cognitive functions (e.g., language and executive functions). Such cognitive effects might indirectly improve other health-related measures such as quality of life and independence in daily living activities, and reduce the burden of caregivers.

One of the main strengths of the present study is its home-based nature, allowing patients to receive treatment in the comfort and familiarity of their own home, reducing distress and costs associated with institutional-based care, and increasing patients’ compliance. This aspect is crucial especially in the case of interventions, such as tACS, requiring multiple sessions and long-term commitment. The use of blood-based AD biomarkers, amyloid-PET, and MRI also enables a comprehensive evaluation of the effects of γ-tACS. Moreover, our study design will allow us to assess both the short-term (i.e., right after the conclusion of the intervention) and the long-term (i.e., 8 weeks after the conclusion of the intervention) efficacy of γ-tACS and to determine whether the duration of the intervention (i.e., 8 weeks vs 16 weeks) affects the long-term efficacy.

## Conclusion

If the expected results are achieved, affordable home-based treatments based on daily γ-tACS sessions might become a reality for the treatment of AD patients and may be implemented alone or in combination with other therapies (e.g., cognitive stimulation or cognitive enhancing drugs) for a synergistic effect.

## Data Availability

Not applicable.

## Abbreviations

AD: Alzheimer’s disease
tACS: Transcranial alternating current stimulation
FDA: Food and Drug Administration
NIBS: Non-invasive brain stimulation
DMN: Default mode network
EEG: Electroencephalogram
TMS: Transcranial magnetic stimulation
MRI: Magnetic resonance imaging
PET: Positron emission tomography
CDR: Clinical Dementia Rating
SAI: Short-latency afferent inhibition
APOE: Apolipoprotein E
MPRAGE: Magnetization-prepared rapid gradient echo
rs-fMRI: Resting-state functional magnetic resonance imaging
ASL: Arterial spin labeling
CBF: Cerebral blood flow
REDCap: Research Electronic Data Capture
